# The Initial Course of IL1β, IL-6, IL-8, IL-10, IL-12, IFN-γ and TNF-α with Regard to Severity Grade in Acute Pancreatitis

**DOI:** 10.3390/biom11040591

**Published:** 2021-04-17

**Authors:** Hanna Sternby, Hannes Hartman, Henrik Thorlacius, Sara Regnér

**Affiliations:** Section for Surgery, Department of Clinical Sciences, Malmö, Lund University, Skane University Hospital, 20502 Malmö, Sweden; hannasternby@gmail.com (H.S.); hannes.hartman@med.lu.se (H.H.); henrik.thorlacius@med.lu.se (H.T.)

**Keywords:** biomarkers, interleukins, chronology, severity, acute pancreatitis, prognostic markers

## Abstract

Clinical reports on early immune dysregulation in acute pancreatitis (AP) are scarce. Herein we investigate the initial temporal development of selected biomarkers. Blood samples were taken at 0–24 and 25–48 h after onsets of AP were acquired. Mean values and temporal intermediate difference (delta-values) of IL-1β, IL-6, IL-8, IL-10, IL-12, IFN-γ and TNF-α were calculated. Differences between severity groups, predictive capacity of the biomarkers and association with severe disease were analyzed. Paired comparison of samples (n = 115) taken at 0–24 and 25–48 h after onsets of AP showed a change over time for IL-1β, IL-6, IL-8 and IL-10 (*p* < 0.05) and a significant difference between severity groups after 24 h. In ROC-analysis an IL-6 cut-off level of 196.6 pg/mL could differentiate severe AP (sensitivity 81.9, specificity 91.3). The delta-values of IL-1β and IL-6 were significantly associated with severe outcomes (odds ratios 1.085 and 1.002, respectively). Data of this work demonstrate a distinct change in IL-1β, IL-8, IL-10 and IL-6 over the first 48 h after onset of AP. The temporal development of biomarkers can assist in the early stratification of the disease. Herein IL-1β and IL-6 were associated with severe disease, however the prognostic capacity of investigated biomarkers is low.

## 1. Introduction

Acute pancreatitis (AP) is a common disease that causes significant burden on health care systems [1,2]. Approximately 30% of the patients develop a systemic course implicating extensive inflammatory reaction and organ dysfunction. The consequences for the patients are long-term hospitalization, substantial morbidity and high risk of death. Early differentiation between mild and severe AP is thus of major importance. During the last decades numerous studies have been conducted in the search for predictive biomarkers and clinical scoring systems. Recent international cohort studies present novel tools regarding both severity differentiation and management of AP [3,4]. However, despite developed frameworks and promising laboratory results a reliable prognostic method still does not exist in general clinical practice [5,6,7].

It is generally recognized that regardless of initiating etiology, a number of pro- and anti-inflammatory mediators are rapidly released during the onset of the disease, causing a local inflammatory response [8,9]. Amplification into systemic involvement results in a general inflammatory reaction with single or multiple organ dysfunction. Additionally, pancreatic enzyme exudation into the peripancreatic adipose tissue triggers adipose lipolysis leading to a release of toxic fatty acids [10]. The interstitial damage drives both cytokine production and eventually massive secretion resulting in a systemic inflammatory reaction and ultimately organ failure.

To be clinically relevant, a prognostic biomarker in AP needs to have certain characteristics including early release and increase during the most initial phase of the disease. It also has to be accessible in the everyday clinical situation. Previous studies have demonstrated tumor necrosis factor-alpha (TNF-α) and interleukin-1β (IL-1β) to be key regulators of the pro-inflammatory response in AP by initiating the reaction to acinar cell injury. Both are also mediators in the production and release of interleukin-6 (IL-6) inter alia [5]. TNF-α is however an unsteady predictive marker as it is promptly purged from the blood by the liver [11]. IL-6 is, by inducing the synthesis of acute-phase proteins, of central importance for the transition into a systemic reaction whereas interleukin-10 (IL-10) levels represent the immune suppressive phase [12,13,14]. Interleukin-8 (IL-8) is a chemokine with aggressive capability to, as a secondary activator, attract neutrophils augmenting the cytokine storm leading to systemic reaction and organ failure [5,15]. Interleukin-12 (IL-12), as well as interferon gamma (IFN-γ), are additional mediators of the inflammatory cascade, although their roles in AP is still unclear.

The pathogenic course might alter rapidly with subsequent clinical deterioration of the patient. The exact roles of local or systemic disease regulators are as of yet unknown and further understanding of the immunological development in AP is required. The majority of studies regarding the initial pathophysiological events in AP have been conducted through experimental animal models [1,16,17,18]. However, implementation of basic science results into clinical practice may not be completely feasible and biomarker studies on AP have, in general, not been clinically reproducible.

Previous findings by our group indicate a need for a more precise examination of the temporal development in AP [19,20]. Due to the rapid immunological changes, we theorize that information on time from onset of symptoms to blood sample is of importance and not presenting exact time intervals might introduce bias and diverging results. Additionally, in precedent clinical studies the results are to a large extent based on former AP classifications and not the revised Atlanta classification [21]. Thus, to obtain clinically relevant results we aimed to, in a routine clinical setting, investigate the initial inflammatory course of AP using established biomarkers and the most recent AP classification. We also hypothesized that a more precise knowledge on the change in biomarker level over time would be essential when analyzing their predictive capacity.

## 2. Materials and Methods

### 2.1. Patients and Study Design

A previously established cohort prospectively and consecutively included patients >18 years with AP admitted to Skåne university hospital, Malmö, from January 2010 to September 2013 [19,20]. For the diagnose of AP 2 out of 3 criteria needed to be fulfilled: (1) acute characteristic upper abdominal pain, (2) serum amylase ≥3 times the upper limit or (3) characteristic findings of AP on computed tomography scan, abdominal ultrasound or magnetic resonance imaging. Only patients admitted within 72 h from onset of disease were included. The patients were retrospectively classified as having mild, moderately severe or severe AP according to the revised Atlanta classification of 2012 [21]. Clinical data, including exact time for onset of pain and validated questions on etiology, were obtained from the patients upon inclusion and retrospectively through review of medical notes. Onset of pain was considered equal to onset of disease. 

The study was approved by the regional ethics committee at Lund University (2009/415). Oral and written consent was provided from all patients prior to inclusion. The study protocol conforms to the ethical guidelines of the 1975 Declaration of Helsinki (6th revision, 2008).

### 2.2. Blood Samples and Biomarkers

Plasma samples were collected upon admission and daily for the following 2 days. Exact time for each blood sampling was registered. The blood samples were collected in plasma separator tubes (containing Lithium-Heparin gel), centrifuged (2000 rounds, 25 °C, 10 min) and stored at −80 °C until analyzed. IFN-γ, IL-1β, IL-6, IL-8, IL-10, IL-12 and TNF-α, were analyzed using human proinflammatory 7-plex ultrasensitive kit (K15008C, Meso Scale Diagnostics LLC, Rockville, MD, USA). Analyses were assessed according to the manufacturer’s instructions. For comparison, admission data (0–24 h) on white blood cells (WBC, ×10^9^/L) and procalcitonin as well as c-reactive protein (CRP, mg/L) data from 0–24 h and 25–48 h after onset of disease were collected. WBC, procalcitonin and CRP were analyzed in accordance with certified standard analysis at the department of Clinical Chemistry, Skåne University Hospital Malmö (ISO 15189:2012, accreditation number 1309).

### 2.3. Statistical Analysis

From the original cohort solely patients with an admission sample taken at 0–24 h and a second sample at 25–48 h after onset of pain were included in analysis. For continuous data, comparison between two groups, Mann-Whitney *U* test was used and *p* < 0.05 was considered statistically significant. Differences in biomarker levels between paired samples taken at 0–24 h and 25–48 h were analyzed using Wilcoxons test.

Mean values for all biomarkers were calculated at 0–24 h and 25–48 h for each severity category. The difference between the mean values of 0–24 h and 25–48 h was denominated delta-value. Variations between delta-values of each severity group were analyzed using Mann-Whitney *U* test. 

ROC-curves (with severe AP as outcome) were performed for the acquirement of area under curve (AUC) and cut-off levels for each delta-value. The individual cut-off levels were investigated for sensibility, specificity, positive predictive value (PPV) and negative predictive value (NPV). For further analysis of the association between delta-values and disease severity univariate and multivariate (adjusted for age and gender) logistic regression analysis were performed.

All statistical analysis was executed using IBM SPSS Statistics for Windows, version 26, Armonk, NY:IBM corp.

## 3. Results

### 3.1. Patient Characteristics

During a period of 3.5 years (2010–2013) 232 patients with AP were consecutively enrolled [19,20]. Within this established cohort 115 patients had an admission sample taken at 0–24 h after onset of disease as well as a second sample taken between 25–48 h into the course of AP. Clinical characteristics of this group are presented in Table 1.

Among the 115 patients 71 (61.7%) developed mild, 33 (28.7%) moderately severe and 11 (9.6%) severe AP. The median age was 65 years, and the group with severe AP was older (median 76 years) compared to the patients with mild and moderately severe AP, however the difference was not significant (*p* = 0.102). Additionally there were no substantial variations in genders or body mass index between severity groups. A majority of the patients (51.3%) had biliary etiology of AP, although numbers were statistically lower among patients with severe AP (27.3%, *p* = 0.035). Significant differences were also found between the mild and severe groups for alcohol misuse and idiopathic etiologies. There was no variation between severity grades in median time from onset of pain to arrival at the hospital.

### 3.2. Biomarkers

Table 2 presents the mean values of each biomarker at 0–24 and 25–48 h separated into severity groups.

Significant alterations were found for IL-8, IL-10 and TNF-α in the mild group, for IL-1β and IL-6 in the moderately severe group and finally for IL-1β, IL-6 and IFN-γ in the severe group. In Table 3 the mean values of each severity group have been compared using the data from Table 2 and the results are presented as *p*-values. The variations were most evident on day two (25–48 h) where significant differences were found between all grades of severity for IL-1β, IL-6, IL-8 and IL-10.

For comparison data on WBC, procalcitonin and CRP were collected. Mean values (0–24 h) for WBC were; 11.7 ± 4.5 (mild AP), 12.9 ± 5.8 (moderately severe AP) and 16.8 ± 4.1 (severe AP). The difference between severity groups was significant (*p* = 0.01) when comparing the severe AP group with the mild and moderately severe groups. Mean values (0–24) for procalcitonin were; 1.86 ± 3.9 (mild AP), 1.46 ± 3.1 (moderately severe AP) and 0.78±1.2 (severe AP). No significant differences between the groups were found. Mean values for CRP on 0–24 h were; 44.9 ± 57.1 (mild AP), 90.3 ± 94.3 (moderately severe AP), 170 ± 110.6 (severe AP) and on 25–48 h; 115.8 ± 95.1 (mild AP), 264.0 ± 126.0 (moderately severe AP) and 302.3 ± 147.0 (severe AP). All CRP groups differed significantly; mild AP versus moderately and severe AP (0–24 h, *p* = 0.0001 and 25–48 h, *p* = 0.001) as well as severe AP versus the mild and moderately severe groups (0–24 h, *p* = 0.0001 and 25–48 h, *p* = 0.005).

The difference between the first and second mean value (delta-value) represents the change over time in biomarker level. The delta-values of the individual biomarkers and disparities between severity grades are presented in Table 4. For all biomarkers except TNF-α, significant difference was found when comparing the groups with mild and severe AP. Only for IL-6 did the delta-values varied significantly between all severity groups. 

The delta-values were further analyzed through ROC-curves presenting an area under the curve and cut-off level with corresponding sensitivity, specificity, positive and negative predictive value for each biomarker (data presented as Table S1 and Figure S1 in Supplementary Material). In this analysis, the best predictive capacity was found for IL-6 with a cut-off delta-value of 196.6 giving an area under the curve of 0.882 with corresponding sensibility and sensibility of 81.8 and 91.3.

The association between delta-values and severe AP was also investigated in a multivariate logistic regression model adjusted for age and gender. In this analysis, only the delta-values of IL-1β and IL-6 demonstrated a significant correlation with severe AP with odds ratios of 1.085 (*p* = 0.011, CI 1.019–1.155) and 1.002 (*p* = 0.033, CI 1.000–1.003), respectively.

## 4. Discussion

AP is a disease with diverse clinical attributes and miscellaneous pathophysiological profile and there is an urgent need to improve our understanding of this inflammatory state [1,22]. Herein we aimed to investigate the initial temporal development of established biomarkers of the inflammatory cascade in AP. Well-known cytokines reflecting different phases of the early pathophysiological process were selected and the analysis was based on specific time frames.

Correlation between disease severity and levels of the cytokines IL-1β, IL-6, IL-8 and IL-10 has been repeatedly demonstrated [13,23,24,25,26,27]. In accordance with previous reports, the present study shows a raise in IL-1β during the first hours after onset of disease, with subsequent releases of IL-6, IL-8 and the anti-inflammatory IL-10 [9,13]. However, clinical investigations on the early (<48 h after onset of pain) development of these biomarkers are deficient with only a few studies published [12,24,27,28]. Additionally, exact time interval from onset of pain to sample collection was rarely reported and thus not included in analysis [6,29]. As AP is a disease where alterations in immune response occur rapidly a difference in hours is likely to matter. Duarte-Rojo et al. presented improved predictive capacity and clinical usefulness of IL-6 and IL-10 when separating samples into strict time intervals [27].

The mean values of IL-1β, IL-6, IL-8, IL-10, IL-12, IFN-γ and TNF-α at 0–24 and 25–48 h after onset of pain describes the initial inflammatory course of the disease. Both IL-1β and IL-6 demonstrate a significant increase over time within the moderately severe and severe groups. In recent reviews and meta-analyses IL-6 was found to be superior in early prediction of moderate and severe AP [5,10,29]. A majority of the biomarkers in our study showed what appeared to be distinct changes in mean values from day one to day two (Table 2). However, the differences were not statistically significant in analysis. This result is due to all severity groups containing both high and low responders, reflecting the large inter-individual variation in the pathophysiology of AP. On an individual level, all biomarker levels in the group who developed severe disease had increased after 24 h. For comparison, data on the well explored biomarkers CRP, WBC and procalcitonin were presented. At 0–24 h CRP differed in all AP severity groups, whereas no variation was found for procalcitonin and only the patients who developed severe AP had a distinctly higher (*p* = 0.01) mean level of WBC upon admission. These biomarkers have repeatedly been associated with severe disease although their predictive capacity is not deemed sufficiently strong [5,6,15,29]. 

Table 4 demonstrates whether the change over time in biomarker mean value (delta-values) differs between the severity groups. From our results there appears to be an evident variation in temporal development when comparing the delta-values of the mild and severe groups. Again the standard deviation values (±SD) highlight the individual diversity of the disease. In ROC-curve analysis of the delta-values we found high negative predictive values for all cut-offs (Supplementary Table S1) indicating that it could be of interest to further investigate the role of these biomarkers regarding the differentiation of patients with mild AP.

We have previously, using the same cohort, investigated the predictive capacity of biomarkers in AP [19,20]. The first study presented results based on the Atlanta classification of 1992 and preset cut-off values were used in analysis. In the second we investigated associations between mild AP and biomarkers. Herein we aimed to, using an exploratory methodology, describe the initial course of biomarkers in AP with regard to all severity groups. We based the analysis on exact time frames including the first days after onset of disease. Our main findings are that the mean values of each severity group differ significantly for IL-1β, IL-6, IL-8 and IL-10 after 24 h. Additionally, the rise in mean value between 0–24 h to 25–48 h (delta-value) is most evident for IL-6.

To the best of our knowledge, this is the first clinical study investigating the early temporal course of biomarkers using the revised Atlanta classification. In some aspects our results differ from previous works, however direct comparisons are difficult to make as earlier studies differ significantly in set-up, primary outcome and severity classification [29,30]. The strength of this study is its prospective setup with consecutively enrolled patients. Acquisition of blood samples together with knowledge on the exact amount of hours passed since onset of pain provides important temporal information of the initial pathophysiology of AP.

This work has several limitations. We used blood samples from an established cohort of 232 patients, however only 115 of these had a first sample taken at 0–24 h after onset of pain and a second sample taken at 25–48 h. Consequently the number of patients with severe AP was low (n = 11), type two error was thus statistically possible and the study was likely under-powered for the investigation of associations between biomarkers levels and severe AP. The low number of patients with severe disease might also have been reflected by the fact that we found no differences in BMI or age between severity groups.

In conclusion, our data show that the difference in mean values between severity groups is more evident 24 h after onset of disease (Table 3). All investigated biomarkers appeared to change distinctly over time (Table 2) and the delta-values varied significantly for all biomarkers but TNF-α when comparing the mild and severe AP groups (Table 4). We also found a large inter-individual variation in biomarker levels between patients within the same severity group, reflecting the complex immunologic condition of the disease. The information presented here has previously not been demonstrated using the revised Atlanta classification and from a clinical angle our results indicate that IL-1β, IL-6, IL-8 and IL-10 are the most relevant biomarkers for early differentiation of severity grades. It is also of our opinion that a more frequent application of precise time frames could further advance the understanding of the immune dysregulation in AP. Detailed comprehension of the pathophysiological changes is essential for the development of therapeutic management and more clinical research has been requested to widen this knowledge [29,30]. Even though the numbers of patients in our study, especially those with severe disease, are small, and the results require further studying and confirmation in a large prospective study, our data indicate that some of the investigated biomarkers have the potential to be of clinical value in future AP stratification.

## Figures and Tables

**Table 1:** Characteristics of patients.

Parameters	All n = 115	MAP n = 71 (61.7%)	MSAP n = 33 (28.7%)	SAP n = 11 (9.6%)	*p*-Value
^¶^ **Gender**					
male	57 (49.6%)	35 (49.3%)	17 (51.5%)	5 (45.5%)	0.545
female	58 (50.4%)	36 (50.7%)	16 (48.5%)	6 (54.4%)	0.621
* Age (years)	65 (20–97) IQR 52–78	63 (20–97) IQR 52–78	65 (29–92) IQR 53–75	76 (31–89) IQR 63–81	0.102
* BMI (kg/m^2^)	25.7 (13.6–47) IQR 23.1–30.4	25.1 (16.0–45.4) IQR 22.6–29.1	27.5 (13.6–40.2) IQR 24.3–32.1	25.5 (21.9–47) IQR 24.2–29.4	0.723
^¶^ **Etiology**					
Biliary	59 (51.3%)	39 (54.9%)	17 (51.5%)	3 (27.3%)	0.035
Alcohol	24 (20.9%)	12 (16.9%)	9 (27.3%)	3 (27.3%)	0.049
Other	18 (15.7%)	13 (18.3%)	3 (9.1%)	2 (18.2%)	0.322
Idiopathic	14 (12.2%)	7 (9.9%)	4 (12.1%)	3 (27.3%)	0.047
* Hours from onset to **admission**	12 (0–24) IQR 4–19	11 (0–24) IQR 4–20	12 (0–24) IQR 5–20	6 (0–21) IQR 1–16	0.178
^¶^ **ICU**	12 (10.4%)	0	4 (12.1%)	8 (72.2%)	<0.001
^¶^ **Organ failure**	22 (19.1%)	0	11 (33.3%)	11 (100%)	<0.001
^¶^ **Mortality**	5 (4.3%)	0	0	5 (45.5%)	<0.001

^¶^ n (%); * Values in median (range). MAP = mild acute pancreatitis; MSAP = moderately severe acute pancreatitis; SAP = severe acute pancreatitis; IQR = inter quartile range; BMI = body mass index; ICU = admission to the intensive care unit. Other etiologies = post-ercp AP + AP due to pancreatic and periampullary tumors + drug-induced AP + AP due to benign strictures.

**Table 2:** Mean values of biomarkers at 0–24 and 25–48 h after onset of disease.

	MAP (n *=* 71)			MSAP (n *=* 33)			SAP (n *=* 11)		
	0–24 h	25–48 h	*p-*Value	0–24 h	25–48 h	*p-*Value	0–24 h	25–48 h	*p-*Value
**IL-1** **β**	2.27(±0.6)	1.9 (±0.28)	0.264	2.4 (±0.65)	5.3 (±1.3)	<0.001	4.0 (±0.9)	15.7 (±6.8)	0.013
**IL-6**	245.1 (±88.4)	179.3 (±72.4)	0.683	351.6 (±215.2)	190.1 (±34.6)	0.01	466.8 (±260.4)	1035.3 (±405.6)	0.004
**IL-8**	189.3 (±56.9)	81.3 (±18.3)	<0.001	166.6 (±54.7)	92.4 (±12.8)	0.611	279.6 (±91.0)	727.2 (±390.2)	0.324
**IL-10**	179.1 (±80.6)	53.4 (±17.1)	<0.001	174.1 (±100.7)	24.6 (±6.0)	0.081	123.5 (±50.1)	705.7 (±428.2)	0.102
**IL-12**	88.3 (±67.9)	84.9 (±68.4)	0.101	18.0 (±15.7)	16.6 (±12.5)	0.721	3.5 (±2.7)	14.6 (±8.2)	0.062
**IFN-** **γ**	15.6 (±5.2)	11.6 (±3.4)	0.782	11.4 (±6.4)	20.3 (±16.5)	0.178	6.3 (±2.1)	22.5 (±10.8)	0.004
**TNF-** **α**	24.9 (±12.9)	11.1 (±2.3)	0.016	23.8 (±11.1)	11.1 (±2.3)	0.254	23.3 (±11.9)	14.0 (±5.2)	0.983

MAP = mild acute pancreatitis; MSAP = moderately severe acute pancreatitis; SAP = severe acute pancreatitis. Values are in mean (±SD); All biomarker units are in pg/mL.

**Table 3:** Differences, describes as *p*-values, in mean values of individual biomarkers.

	0–24 h			25–48 h		
	MAP-MSAP	MSAP-SAP	MAP-SAP	MAP-MSAP	MSAP-SAP	MAP-SAP
IL-1β	0.390	0.023	0.003	<0.001	0.05	<0.001
IL-6	0.128	0.237	0.039	<0.001	<0.001	<0.001
IL-8	0.701	0.105	0.058	0.003	0.015	<0.001
IL-10	0.261	0.145	0.042	0.031	0.002	<0.001
IL-12	0.595	0.437	0.259	0.593	0.728	0.858
IFN-γ	0.310	0.422	0.658	0.385	0.009	0.028
TNF-α	0.072	0.206	0.951	0.028	0.153	0.610

Mean values used in this analysis are equal to those presented in Table 2. MAP = mild acute pancreatitis; MSAP = moderately severe acute pancreatitis; SAP = severe acute pancreatitis

**Table 4:** Delta-values and statistical differences between severity groups.

	Delta-Values			*p* Values		
	MAP n = 71	MSAP n = 33	SAP n = 11	MAP-MSAP	MSAP-SAP	SAP-MAP
**IL-1** **β**	0.32 (±0.49)	2.8 (±1.3)	11.7(±6.3)	<0.001	0.238	0.001
**IL-6**	65.7 (±79.9)	160.1 (±212.5)	569.1 (±171.2)	0.001	0.001	<0.001
**IL-8**	108.0 (±44.9)	74.1 (±53.2)	447.6 (±305.1)	0.038	0.196	0.014
**IL-10**	125.2 (±70.3)	149.5 (±100.6)	582.2 (±385.2)	0.268	0.017	0.004
**IL-12**	4.6 (±3.2)	1.4 (±20.5)	11.1 (±8.3)	0.571	0.093	0.036
**IFN-** **γ**	4.3 (±2.9)	8.9 (±20.5)	16.1 (±8.9)	0.545	0.004	0.001
**TNF-** **α**	14.27 (±11.7)	15.5 (±10.7)	9.3 (±6.8)	0.737	0.612	0.480

MAP = mild acute pancreatitis; MSAP = moderately severe acute pancreatitis; SAP = severe acute pancreatitis. Values are in mean (±SD); All biomarker units are in pg/mL.

## Data Availability

Data supporting reported results is available upon request through contact with the corresponding author.

## Supplementary Materials

The following are available online at https://www.mdpi.com/article/10.3390/biom11040591/s1, Figure S1: ROC-curves of delta-values; Table S1: Cut-offs for delta-values with regard to severe disease
