# Supplementary material for: The Initial Course of IL1β, IL-6, IL-8, IL-10, IL-12, IFN-γ and TNF-α with Regard to Severity Grade in Acute Pancreatitis
Source: Biomolecules. 2021 Apr 17;11(4):591. doi: 10.3390/biom11040591 (PMC8073083; doi:10.3390/biom11040591)
Supplement: Supplementary file 1 [file biomolecules-11-00591-s001.zip › Supplementary files.pdf]

Figure 1. ROC-curves of delta-values

A

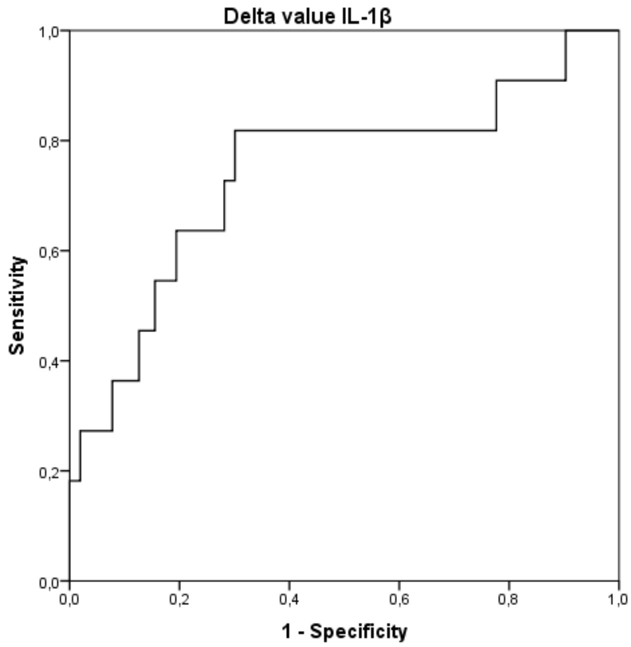

AUC: 0.742

B

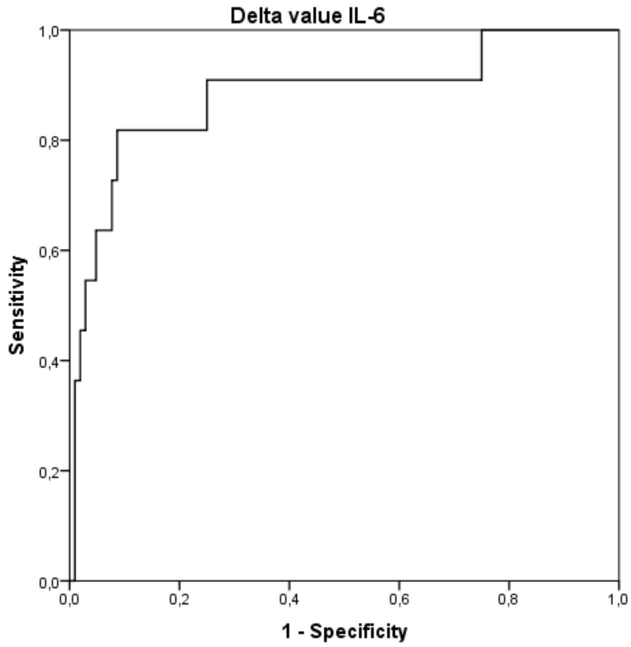

AUC: 0.888

C

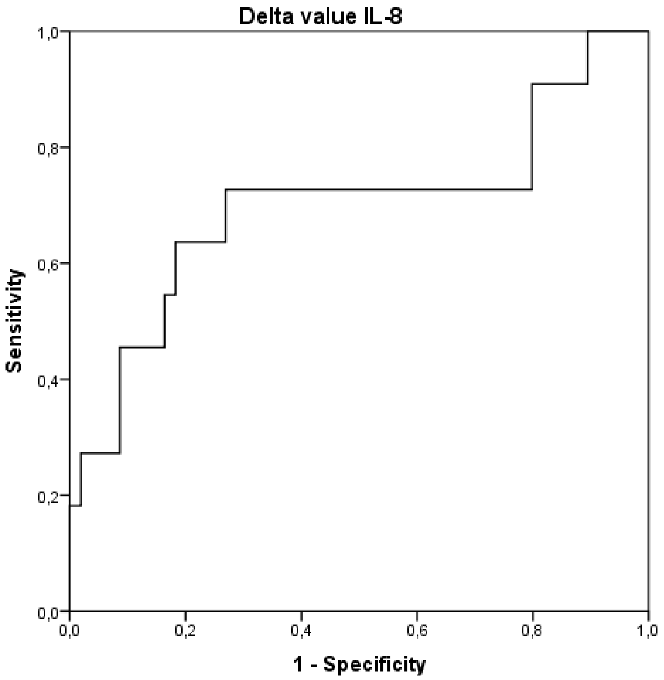

AUC: 0.700

D

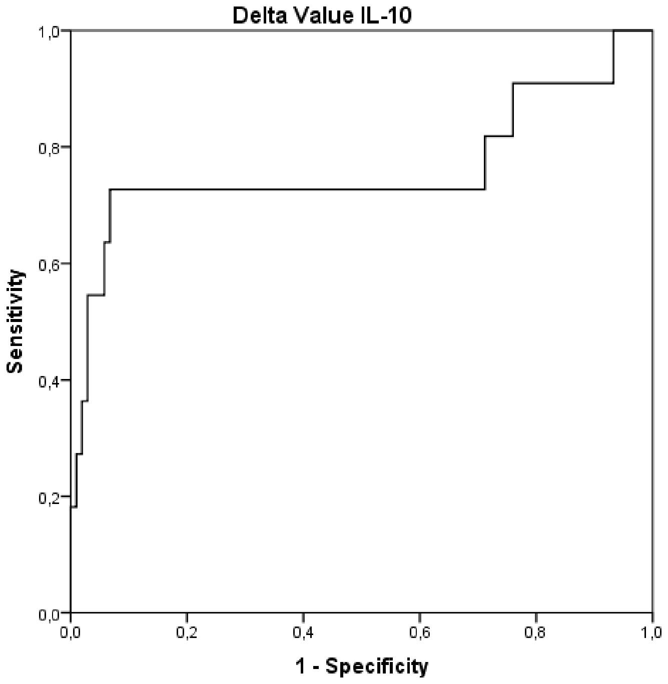

AUC: 0.762

E

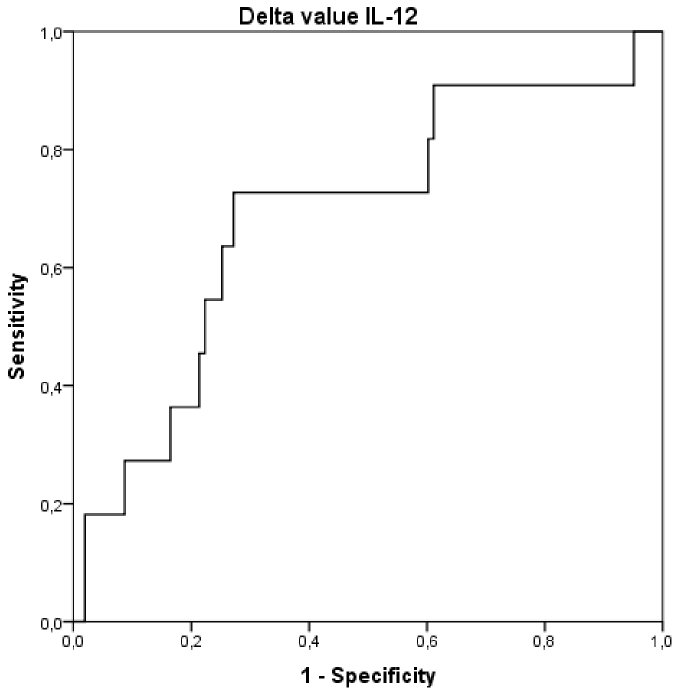

AUC: 0.689

F

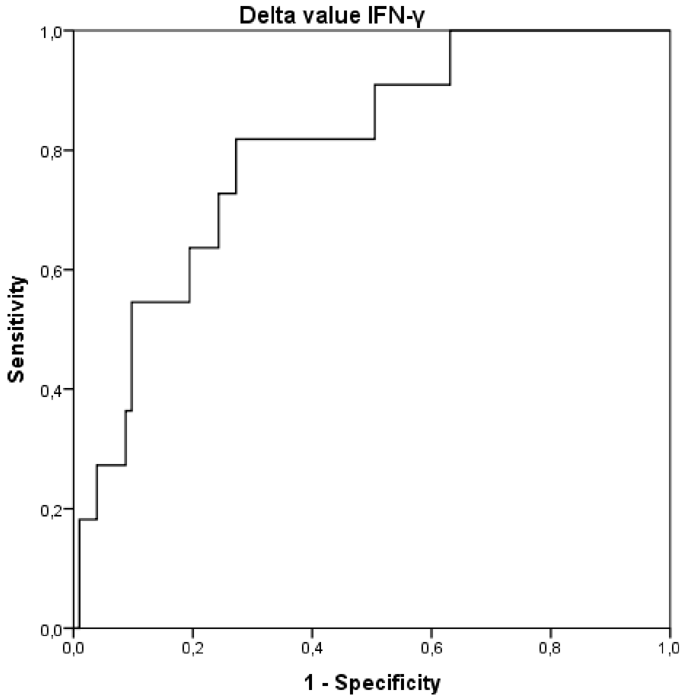

AUC: 0.801

G

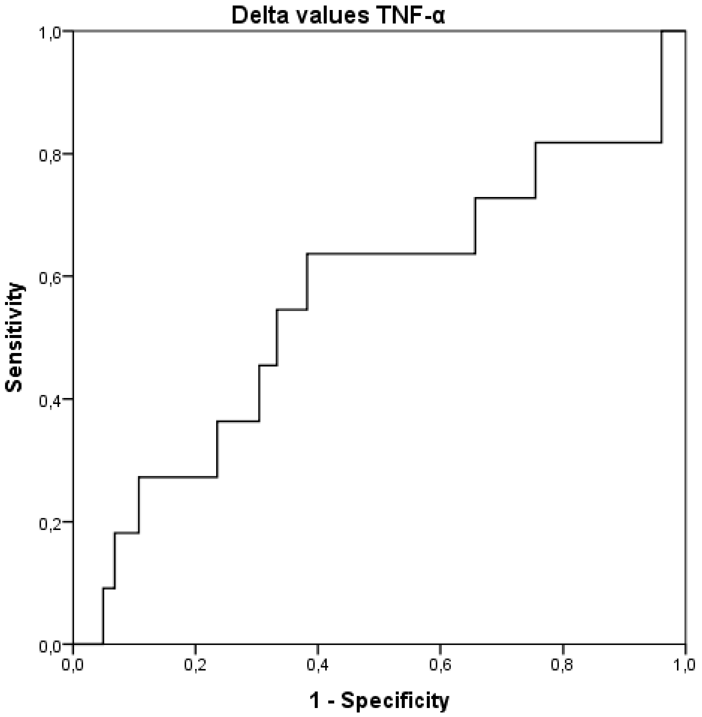

AUC: 0.562

Figure legends

ROC-curves and Area Under Curves (AUCs) of delta-values for IL_1β (A), IL-6 (B), IL-8(C), IL-10 (D), IL-12 (E), IFN-γ (F) and TNF_α (G).

Table 1 Cut-offs for delta-values with regard to severe disease

	Cut-off	AUC	Sensitivity	Specificity	PPV	NPV
**IL-1β**	0,944	0.742	81.8	69.9	0.196	0.971
**IL-6**	196.6	0.882	81.8	91.3	0.5	0.98
**IL-8**	1.46	0.700	72.7	73.1	0.22	0.96
**IL-10**	9.54	0.762	72.7	93.3	0.533	0.970
**IL-12**	0.15	0.689	72.7	72.8	0.22	0.96
**IFN-γ**	0.77	0.801	81.8	72.8	0.24	0.97
**TNF-α**	0.07	0.562	63.6	61.8	0.15	0.94

AUC = area under curve; PPV = positive predictive value; NPV = negative predictive value

All biomarker units are in pg/ml
